# Referral patterns and outcomes of children who failed Modified Checklist for Autism in Toddlers screening during routine health screening at maternal and child health clinics in the northeast district of Penang: A retrospective cohort study

**DOI:** 10.51866/oa.378

**Published:** 2024-02-07

**Authors:** Ranjini Ambigapathy, Sathyabama Ramachandram, Fairuz Fadzilah Rahim

**Affiliations:** 1 MBBS, M.MED (FAM MED), Klinik Kesihatan Sungai Dua (Timur Laut), Lot 2544, Mukim 13, Gelugor, Pulau Pinang, Malaysia. Email: ranjini_ambigapathy@yahoo.com; 2 MBBS, MRCPCH, FRCPCH, Department of Paediatrics, Penang General Hospital, Jalan Residensi Georgetown Penang, Malaysia.; 3 BScBiomed, MScMedStats, Department of Public Health Medicine, RCSI & UCD Malaysia Campus 4, Sepoy Lines Road, George Town, Penang, Malaysia.

**Keywords:** Early detection of disease, Autism spectrum disorder, Primary health care, Mass screening, Referral and consultation

## Abstract

**Introduction:**

Autism spectrum disorder (ASD) is a developmental disability that causes significant social, communication and behavioural challenges. The Modified Checklist for Autism in Toddlers (M-CHAT) is a parent-administered screening questionnaire for ASD used at 18 and 36 months of age. This study aimed to determine the outcomes of children who failed M-CHAT screening during routine health screening at maternal and child health clinics in northeast district, Penang and the prevalence of ASD among those with a final diagnosis.

**Methods:**

This retrospective cohort study was conducted at 12 maternal and child health clinics. All children who failed M-CHAT screening at 18 and 36 months from January 2017 to December 2021 and received a final diagnosis before 31 March 2022 were recruited. All information required was recorded in a data collection form and analysed using SPSS. Multiple logistic regression was performed to assess the association between the factors and ASD status.

**Results:**

Eighty-two children failed M-CHAT screening. Fifty children did not receive a final diagnosis. Among 32 children who received a final diagnosis, 25 were diagnosed with ASD (78.1%). Among the children who underwent M-CHAT screening, the odds of having ASD increased by a factor of 1.2 for every 1-unit increase in age at final diagnosis.

**Conclusion:**

In northeast district, Penang, more than half of children who fail M-CHAT screening have no final diagnosis. The specificity of the M-CHAT is 78.1% among children with a final diagnosis of ASD. The age at final diagnosis is positively associated with the diagnosis of ASD.

## Introduction

Autism spectrum disorder (ASD) is a developmental disability that causes significant social, communication and behavioural challenges. Early identification of ASD leads to appropriate intervention and better outcomes, with higher potential for enrolment into mainstream education.^1-4^

In 2020, 1 in 36 children aged 8 years was identified to have ASD based on estimates from 11 Autism and Developmental Disabilities Monitoring (ADDM) Network sites across the United States (US; Arizona, Arkansas, California, Georgia, Maryland, Minnesota, Missouri, New Jersey, Tennessee, Utah and Wisconsin).^5^ No local epidemiological study has yet investigated the prevalence of ASD in Malaysia.^6^ However, usage of the Modified Checklist for Autism in Toddlers (M-CHAT) as a screening tool at health clinics by the Ministry of Health Malaysia shows a prevalence of ASD of 1.6 in 1000 children aged 18–36 months.^6^ The M-CHAT is a 23-item parent-administered screening questionnaire recommended to be utilised at 18 and 36 months of age to assess the risk for ASD.^1^ The specificity of the M-CHAT is reported to be as high as 98%, while the sensitivity, specificity and PPV range from 70% to 92%, from 27% to 43% and from 5.8% to 76%, respectively.^2^ The M-CHAT has been incorporated into the Malaysian child health record book. Completed questionnaires, either self-administered by parents or guided by nurses, are then scored and recorded in the child health record book.

Children who fail the screening are referred to medical officers or family medicine specialists at clinics and subsequently to tertiary care settings after assessment.

At present, census on children failing M-CHAT screening is kept by maternal and child health clinic staff in Malaysia. However, to our knowledge, there are no records documenting the outcomes of children who fail M-CHAT screening at primary care facilities after referral to tertiary care centres for diagnosis. Accordingly, this study aimed to determine the outcomes of children who failed M-CHAT screening during routine health screening conducted at 18 and 36 months of age and the prevalence of ASD among those who received a final diagnosis. We also identified possible factors associated with ASD in this cohort.

## Methods

This retrospective cohort study was conducted at 12 maternal and child health clinics in the northeast district of Penang in July 2021.

**Figure 1:**
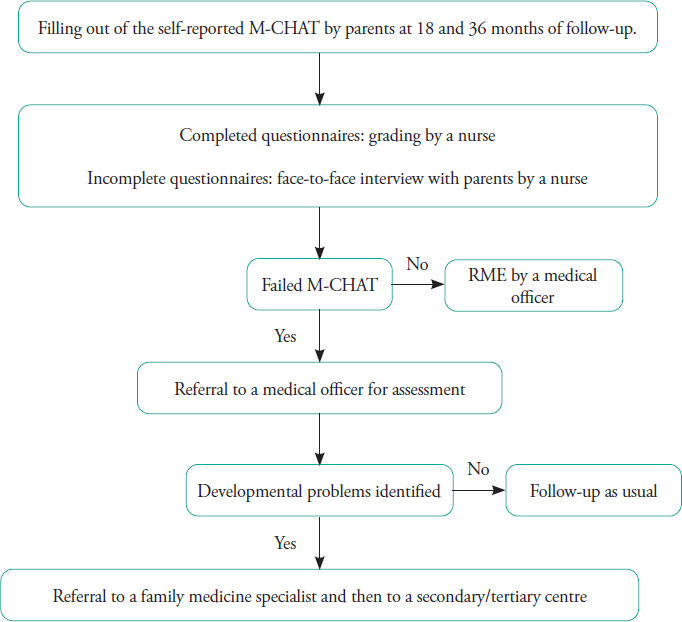
Flowchart of the routine Modified Checklist for Autism in Toddlers (M-CHAT) screening process at the health clinics.

**Figure 2:**
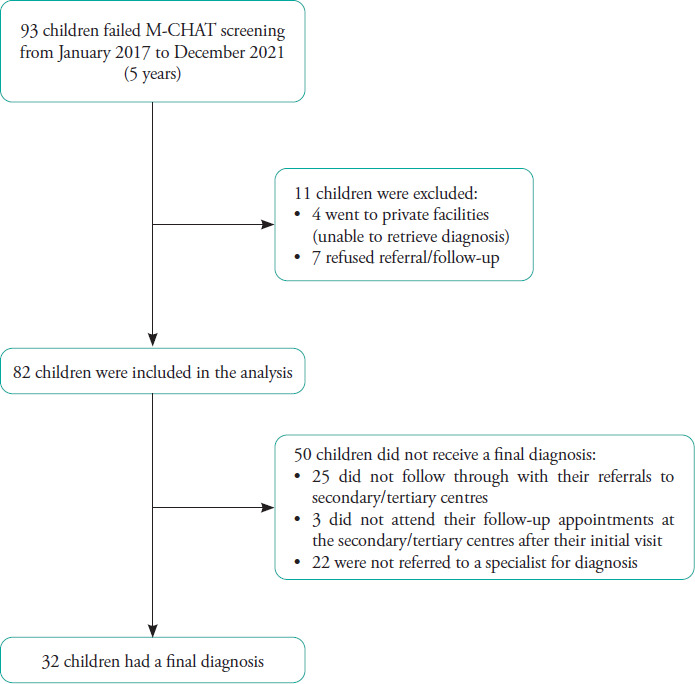
Flowchart of data collection.

All children who failed M-CHAT screening during routine health screening (Figure 1) at 18 and 36 months of age in the past 5 years (from January 2017 to December 2021) and received a final diagnosis before 31 March 2022 were included. A data collection form for demographic characteristics, birth and neonatal history, family history of ASD, final diagnosis and duration from screening to final diagnosis was used. Data of the children who failed M-CHAT screening during the study period were collected from records derived from yearly census at the 12 maternal and child health clinics (Figure 2). The final diagnosis of the children who were referred to tertiary care centres was derived from patient case records at a child development clinic, general paediatric clinic and child psychiatry clinic in Penang Hospital. The data collection forms were numbered from 00 to 82. No names or other identity numbers were used to ensure patient confidentiality. Ethical approval was obtained from the Malaysian Research and Ethics Committee (MREC) with MREC number NMRR ID-22-01251-G4Y.

Parental age at the time of delivery, gestational age, birth weight, mode of delivery, maternal illness, neonatal complications, family history of ASD and other comorbidities were also evaluated to identify possible factors associated with ASD.

Categorical variables were summarised as frequencies and percentages, continuous variables with a normal distribution as means and standard deviations (SDs) and continuous variables with a skewed distribution as medians (interquartile ranges). The final diagnoses were categorised using the Diagnostic and Statistical Manual of Mental Disorders, Fifth Edition. Univariable and multivariable analyses were conducted using logistic regression to test for associations between the variables. Statistical significance was considered at a P-value of <0.05.

## Results

Of the 93 children who failed M-CHAT screening, four opted to go to private facilities, and seven refused further assessment. Ultimately, 82 children were included in this study.

Table 1 shows the demographic characteristics of the children who failed M-CHAT screening. The mean age of both the mothers and fathers at delivery ranged from 30 to 39 years. The mean gestational age was 38.26 weeks (SD=1.84), and the mean birth weight was 3.02 kg (SD=0.47). Most mothers delivered via spontaneous vaginal delivery (59.8%), and about 5% had gestational diabetes mellitus. Around 9% of the children had a family history of ASD. The majority of the children underwent M-CHAT screening at 18 months of age (78.0%). The median duration from review by a secondary or tertiary centre to M-CHAT screening was close to 11 months. The median duration from M-CHAT screening to final diagnosis was about 13 months. The final diagnosis could not be made during the first visit to the secondary or tertiary centres, as more than one visit was required before diagnosis.

**Table 1:** Demographic characteristics of the children who failed Modified Checklist for Autism in Toddlers (M-CHAT) screening (N=82).

Factors	n (%)
Maternal age at delivery	30.16 (5.39)^a^
Paternal age at delivery (n=78)	33.10 (5.77)^a^
Gestational age (week)	38.26(1.84)^a^
Birth weight (kg)	3.02 (0.47)^a^
Mode of delivery	
Assisted delivery^*^	3 (3.7)
Lower-segment caesarean section	30 (36.6)
Spontaneous vaginal delivery	49 (59.8)
Maternal illness during delivery	
Gestational diabetes mellitus	4 (4.9)
Hypertension^**^	2 (2.4)
Hypothyroidism	1 (1-2)
Preeclampsia^#^	1 (1-2)
Unsure	1 (1-2)
Nil	73 (89.0)
Age at M-CHAT screening	
18 months	64 (78.0)
36 months	18 (22.0)
Family history of autism spectrum disorder	
Yes	7 (8.5)
No	73 (89.0)
Unsure	2 (2.4)
Duration from M-CHAT screening to final diagnosis, month (n=29)	13 (14)^b^
Child age at final diagnosis, month (n=32)	36.50 (21)^b^
Duration from review by a secondary/tertiary centre to M-CHAT screening, month (n=30)^c^	10.50 (14)^b^

a Mean (SD)

b Median (IQR)

c Secondary centre: general paediatric clinic, tertiary centre: child development clinic or child psychiatry clinic

* Assisted delivery: vacuum/forceps delivery

** Hypertension during pregnancy: pregnant mother with pre-existing hypertension

# Mother presenting with signs and symptoms of preeclampsia during pregnancy

The majority of the children (87.8%) did not experience complications during delivery. The neonatal complications identified were neurological disorder (8.5%), genetic disorder (2.4%), heart disease (4.9%), neonatal jaundice (14.6%), prematurity (3.7%), global developmental delay (6.1%) and others such as pneumonia, deranged thyroid function, flat foot, neonatal hypoglycaemia, ptosis and resolved sepsis (7.3%).

Table 2 presents the findings of a sub-analysis among the children who received a final diagnosis of ASD. Of these children, 17 (68%) also had global developmental delay/intellectual impairment; one (4%) had attention-deficit hyperactivity disorder; and seven (28%) did not have any other comorbidities. Among the children with ASD with and without comorbidities, 78.3% and 17.4% had a social communication severity of II and III, respectively, while 30.4% and 69.6% had a restricted repetitive behaviour severity of I and II, respectively.

**Table 2:** Diag nosis of ASD among the children who failed M-CHAT screening and received a final diagnosis (n=32).

Final diagnosis	n (%)
ASD among those with a final diagnosis (n=32)	25 (78.1)
Diagnosis of children with confirmed ASD (n=25) ASD with GDD/ID	17 (68)
ASD with ADHD	1 (4)
ASD without comorbidities	7 (28)

GDD/ID, global developmental delay/intellectual disability; ADHD, attention-deficit hyperactivity disorder

Table 3 displays the factors associated with ASD in the multiple logistic regression analysis. Among the children who underwent M-CHAT screening, the odds of having ASD increased by a factor of 1.2 for every 1-unit increase in age at final diagnosis, assuming that all other variables remained constant.

**Table 3:** Multiple logistic regression analysis of the factors associated with ASD (n=32).

Factors	n (%)	Crude OR (95%CI)^b^	P-value^c^	Adjusted OR (95% CI)^c^	P-value^c^
Maternal age at delivery	29.41 (5.84)^a^	1.02(0.88,1.17)	0.833		
Paternal age at delivery	32.42 (6.80)^a^	0.98 (0.87,1.12)	0.794		
Gestational age (week)	38.50(1.32)^a^	1.17 (0.62,2.22)	0.623		
Birth weight (kg)	3.01 (0.31)^a^	12.04 (0.44,330.53)	0.141		
Mode of delivery Assisted delivery LSCS SVD	2(6.3) 12 (37.5) 18 (56.3)	1 2.00 (0.37,10.92) 1.33(0.24,7.34)	0.423 0.741		
Maternal illness during delivery	4(12.5)	0.82 (0.07,9.36)	0.872		
Age at M-CHAT screening 18 months 36 months	23 (71.9) 9 (28.1)	1 2.82 (0.29,27.54)	0.372		
Child age at final diagnosis (month)	36.90 (15.58)^a^	1.16(1.02,1.33)	0.028	1.16(1.01,1.32)	0.032
GDD/ID	20 (62.5)	2.83 (0.51, 15.77)	0.234		

a Mean (SD)

b Simple logistic regression

c Multiple logistic regression using the backward (LR) method (Hosmer and Lemeshow test, P=0.958; Nagelkerke R^2^=0.725; classification table=86.7%). GDD/ID, global developmental delay/intellectual disability

## Discussion

Among the children who failed M-CHAT screening and received a final diagnosis in this study, 78.1% were diagnosed with ASD. The M-CHAT has been proven to have a high specificity of 98%,^6^ as also observed in this study. Accordingly, the M-CHAT is an appropriate and simple screening tool to be used in primary care settings to identify children at risk of ASD.

In the present study, there was a positive association between age at final diagnosis and diagnosis of ASD. This finding is consistent with that of a study conducted in Toronto in 2015, where many siblings of children with ASD were not diagnosed with ASD during M-CHAT screening at 18 months of age but were diagnosed at 36 months of age.^7^ A full diagnostic evaluation at 18 months of age may not be able to identify high-risk children who later receive a diagnosis of ASD owing to the variability in the symptom profile.^7,8^ The American Academy of Pediatrics recommends that all children be screened for ASD during child visits at 18, 24 and 36 months of age and when a parent expresses concern that ASD may be missed during earlier child health visits.^4^

The duration from the final diagnosis to screening in this study was around 13 months, and the age of the children who received their final diagnosis was 3 years (average=36.5 months). Similarly, a study performed in the US showed that the average age at diagnosis was 33.8 months (SD=9.7; range=18–56 months). Conversely, a study conducted in Norway demonstrated that the median age at diagnosis was 4 years,^9^ but many children presented with symptoms much earlier at around 18 months of age.^10,11^ The metaanalysis conducted in 2020 by van ’t Hof et al. in the Netherlands to evaluate the average age at diagnosis across studies published from 2012 to 2019 showed a mean age at diagnosis of 43.18 months among children aged 10 years and below.^12^ Some of the common barriers to early diagnosis often quoted in the literature include a lack of knowledge among parents and healthcare workers, social stigma, dismissal of parents’ initial concerns by healthcare providers and access to ASD services.^13-17^ Other healthcare factors identified are inadequate time for healthcare providers to screen ASD in primary care settings and a lack of trained healthcare providers to perform ASD screening.^13^ Accordingly, the current practice of M-CHAT screening at 18 and 36 months of age is appropriate.

A US study demonstrated that only 31% of children who failed M-CHAT screening were referred to a specialist, while 65% of them completed their referrals.^18^ These findings are in contrast with the current findings: There are differences in practice between our setting and the setting in the US. In our setting, most primary care doctors refer children who fail their M-CHAT screening owing to the referral system in place and adherence to the preexisting local guidelines on ASD. However, the majority of children in this study did not follow through with their referrals (30.5%) and defaulted after their first visit (3.7%). The lack of awareness among parents, the failure to follow through on referrals or a combination of the two and other potential factors could have contributed to the lower rates of referral completion.

In the current study, 26.8% of the children who failed their M-CHAT screening were not referred by their primary care doctors to specialists in tertiary care settings for diagnosis. Most children were referred for intervention to occupational and/or speech therapists. However, among these children, those with undiagnosed ASD may miss out on subspecialty assessment and recommendation. Some factors contributing to delay in referrals could be a lack of training and knowledge of the pre-existing referral system. Yearly training on ASD clinical practice guidelines has been provided to healthcare providers in Penang, emphasising the use of M-CHAT as a screening tool across the state and reinforcing the pre-existing referral pathway in place. Owing to the COVID-19 pandemic, all trainings had been temporarily put on hold, resulting in new staff not being able to receive the training needed. From the end of 2022, training has resumed, and future studies can be conducted to evaluate the effectiveness of the training. In addition to existing training, family medicine specialists with special interest in neurodevelopmental disorders can be provided with options of further subspecialisation or short attachments at tertiary care centres to enable the assessment and management of uncomplicated cases at primary care centres.

A possible measure that can be implemented to address the low rates of referral completion is to monitor the records of patients who are referred. One option that might help is to create a database for children who fail their M-CHAT screening, linking primary and secondary/tertiary healthcare facilities with healthcare staff to ensure that appointments are followed through.

To our knowledge, this study is the first in Malaysia to focus on the outcomes of children who failed their M-CHAT screening at primary care facilities after referral to tertiary care centres. Universal sampling was used to achieve a good representation of children attending the health clinics, making the results generalisable to the study population. However, there are a few limitations of the study. First, the sample size was small. Accordingly, the statistical analysis was limited. A better statistical analysis could have been performed with a larger sample size to determine the factors associated with ASD. Second, the study was conducted in one out of five districts in Penang, which is located in an urban area, and the public tertiary referral centre is located in the district involved in this study. The findings may therefore not be representative of the general population in the community. Three of the districts are located further from the tertiary referral centre (26-50 km), which may affect the findings of this study. Future surveys involving a larger population in the entire Penang state could provide further data on the outcomes of children failing M-CHAT screening.

This study offers a new perspective on the screening and referral processes for children who fail their M-CHAT screening. Substantial effort is put into early screening and referral at primary healthcare facilities because there is enough evidence to support the idea that children who receive behavioural intervention at a younger age have better developmental outcomes than children who do at an older age.^19,20^ However, although developmental delays were identified early, the final diagnosis could not be obtained among more than half of the children in the present study.

## Conclusion

The M-CHAT is proven to be a good screening tool for early detection of ASD among children. However, many children fail M-CHAT screening but do not receive a confirmatory diagnosis owing to parental and healthcare professional-related factors. The importance of early diagnosis and intervention should be emphasised among parents. Networking between different healthcare facilities is essential to ensure that children referred from primary and secondary care centres complete their consultations with specialists at tertiary care centres. Future similar studies should be conducted in other districts in Penang and other states to obtain more accurate and generalisable data. Barriers to the completion of specialty referrals should also be identified, and measures to overcome them should be devised.

## Data sharing statement

No additional data beyond what is presented in this manuscript will be shared.

**How does this paper make a difference in general practice?**

- Modified Checklist for Autism in Toddlers (M-CHAT) screening has long been conducted in primary care settings for children to receive early diagnosis of autism spectrum disorder and early intervention.
- There is no database containing the outcomes of the current screening and referral system.
- This study could serve as a basis for exploring possible challenges and establishing a proper referral pathway to ensure early diagnosis in children failing their M-CHAT screening.
- A proper database linking primary and tertiary care can be created to ensure that children receive early diagnosis.
